# Comparative Analysis of Cell-Associated HIV DNA Levels in Cerebrospinal Fluid and Peripheral Blood by Droplet Digital PCR

**DOI:** 10.1371/journal.pone.0139510

**Published:** 2015-10-02

**Authors:** Michelli Faria de Oliveira, Sara Gianella, Scott Letendre, Konrad Scheffler, Sergei L. Kosakovsky Pond, Davey M. Smith, Matt Strain, Ronald J. Ellis

**Affiliations:** 1 University of California, San Diego, La Jolla, California, United States of America; 2 HIV Neurobehavioral Research Center, University of California, San Diego, San Diego, California, United States of America; 3 Stellenbosch University, Stellenbosch, South Africa; 4 Veterans Affairs San Diego Healthcare System, San Diego, United States of America; University of Pittsburgh Center for Vaccine Research, UNITED STATES

## Abstract

**Background:**

Measurement of HIV DNA-bearing cells in cerebrospinal fluid (CSF) is challenging because few cells are present. We present a novel application of the sensitive droplet digital (dd)PCR in this context.

**Methods:**

We analyzed CSF cell pellets and paired peripheral blood mononuclear cells (PBMC) from 28 subjects, 19 of whom had undetectable HIV RNA (<48 copies/mL) in both compartments. We extracted DNA from PBMC using silica-based columns and used direct lysis on CSF cells. HIV DNA and the host housekeeping gene (RPP30) were measured in CSF and PBMC by (dd)PCR. We compared HIV DNA levels in virally-suppressed and-unsuppressed subgroups and calculated correlations between HIV DNA and RNA levels in both compartments using non-parametric tests.

**Results:**

HIV DNA was detected in 18/28 (64%) CSF cell pellets, including 10/19 (53%) samples with undetectable HIV RNA. HIV DNA levels in CSF cell pellets were not correlated with RPP30 (p = 0.3), but correlated positively with HIV RNA in CSF (p = 0.04) and HIV DNA in PBMC (p = 0.03). Cellular HIV DNA in CSF was detected in comparable levels in HIV RNA-suppressed and unsuppressed subjects (p = 0.14). In contrast, HIV DNA levels in PBMC were significantly lower in HIV RNA-suppressed than in unsuppressed subjects (p = 0.014). Among subjects with detectable HIV DNA in both compartments, HIV DNA levels in CSF were significantly higher than in PBMC (p<0.001).

**Conclusions:**

Despite low mononuclear cell numbers in CSF, HIV DNA was detected in most virally suppressed individuals. In contrast to PBMC, suppressive ART was not associated with lower HIV DNA levels in CSF cells, compared to no ART, perhaps due to poorer ART penetration, slower decay of HIV DNA, or enrichment of HIV DNA-bearing mononuclear cells into the CSF, compared to blood. Future studies should determine what fraction of HIV DNA is replication-competent in CSF leukocytes, compared to PBMC.

## Introduction

HIV enters the central nervous system (CNS) early during the course of infection and establishes a latent HIV reservoir that cannot be eliminated by current antiretroviral therapy (ART) [1]. Previous studies have reported substantial inter-individual differences in the frequencies of cells harboring HIV DNA in blood in relation to duration of HIV infection, time on ART initiation, and multiple host and viral factors [2,3]. Previous rigorous studies have estimated that less than 1% of PBMC contain proviral HIV DNA during ART [2,4], but the frequency of HIV-infected cells in the cerebrospinal fluid (CSF) during virologic suppression on ART is less clear [5,6]. This is important because the CSF is the standard proxy for the immunological and virological dynamics in the CNS [1,7–9], and there are no practical means to work with brain tissues directly *in vivo*. However, evaluation of HIV DNA in CSF collected with lumbar puncture is also limited because it often has low numbers of cells. Generally, the number of leukocytes in CSF is several orders of magnitude smaller than in blood. In particular, CSF leukocytes typically number 5/mm^3^ or less, while the number of leukocytes in blood is ~5000/mm^3^. It is therefore important to develop and use the most sensitive and accurate assays to measure cell associated HIV DNA in CSF samples. To this end, we adapted droplet digital (dd)PCR technology to measure levels of HIV DNA in CSF cell pellets [10]. We then explored associations between HIV DNA levels in CSF and blood in ART-suppressed and non-suppressed HIV-infected subjects.

## Material and Methods

### Study Population and Samples

We evaluated 29 HIV infected subjects from the UCSD HIV Neurobehavioral Research Center (HNRC) cohort. Participants were retrospectively selected based on the availability of peripheral blood mononuclear cells (PBMC) and CSF cell pellets. Participants were selected to represent groups on and off ART, and to span a range of HIV RNA levels in blood and CSF supernatant. Samples with levels of HIV RNA in blood plasma and CSF bellow 48 copies/mL were considered undetectable. The CNS penetration effectiveness (CPE) index for the most recent ART regimen was determined as previously described [11]. Blood CD4+ T-lymphocyte subsets were measured by flow-cytometry (CLIA certified local laboratory). HIV RNA levels were quantified by the Amplicor HIV Monitor Test (Roche Molecular Systems Inc.). We also selected 20 CSF cellular pellets from HIV negative subjects to be used as negative controls for ddPCR. All participants provided appropriate written informed consent and the study was approved by Human Research Protections Program at University of California San Diego.

### Quantification of HIV DNA from PBMC and CSF

Genomic DNA was extracted from PBMC using QIAamp DNA Midi Kit (Qiagen) per manufacturer's protocol. Direct lysis was used for HIV-positive and HIV-negative CSF cell pellets obtained from a volume of 4 mL of CSF from lumbar puncture, as previously described [12]. Levels of HIV DNA were measured by (dd)PCR and reported as the number of copies of HIV DNA per 10^6^ cells, as previously described [10]. The number of ribonuclease P (RPP30) copies detected in each (dd)PCR reaction was used to estimate the number of cells per cellular pellet. Samples of CSF cellular pellets from 20 HIV negative subjects were used as non-template controls (NTC) to determine the rates of false positive droplets in the ddPCR. Lysis buffer and ddPCR assay for these samples were performed following the same procedures as for the HIV infected samples.

### Statistical analysis

Statistical differences between variables measured for the groups of participants with and without detectable HIV RNA levels in blood plasma, and for HIV DNA levels between anatomic compartments (i.e. CSF cell pellet and PBMC) were evaluated using the rank-based Mann-Whitney test. Fisher’s exact test was used to evaluate the differences between the proportions of samples with detectable or undetectable HIV RNA in CSF between groups. We used Kendall’s rank correlation to quantify associations between levels of DNA and RNA in different compartments. All of the statistical tests were performed in R (version 3.1.1, http://www.R-project.org/).

## Results

### Study population

Characteristics of HIV-infected participants are summarized in Table 1. The HIV-positive study cohort included 19 subjects with undetectable HIV RNA in blood plasma (<48 copies/ml), of whom 18 also had undetectable HIV RNA in CSF supernatant. The remaining 9 subjects presented detectable HIV RNA in blood plasma with a median of 3.8 log_10_ HIV RNA (interquartile range [IQR]: 3.5–4.8). The median log_10_ HIV RNA in CSF supernatant was 4.1 (IQR: 3.9–4.4) among 9 subjects with detectable HIV RNA in CSF. The overall median CD4 count was 504 cells/μl (IQR: 362–671) and the CD4 count was not significantly different between subjects with and without detectable HIV RNA in blood plasma. Twenty-one individuals (75%) were on ART at the time of sample collection, eighteen of them were on regimens including a protease inhibitor and three were receiving a combination of 2 nucleoside and 1 non-nucleoside reverse transcriptase inhibitors. One subject was ART-naïve and the remaining 6 subjects had a past history of ART use but were off ART at the time of sample collection. For those on ART at time of sampling and with suppressed HIV RNA levels in blood plasma, the median time of exposure to the most recent ART regimen was 2.4 years (IQR: 1.2–3.4), and the median CPE index for the most recent ART regimen was 7 (IQR: 7–6.7).

**Table 1:** Population characteristics and levels of HIV RNA and DNA in PBMC and CSF.

Characteristic	Undetectable HIV RNA group^c^ (N = 19^d^)	Detectable HIV RNA group^c^ (N = 9)	Mann-Whitney Testp
EDI (years)^a^	12.5 [7.8–16]	18.4 [17.8–21.2]	0.09
CD4 count^a^	506 [350–801]	458.5 [383.3–553.3]	0.58
CPE^a^	7 [6.5–7]	9 [8–10]	0.50
Currently on ART, n (%)	19 [100]	2 [22]	-
Time on ART (years)^a^ ^,^ ^b^	2.4 [1.2–3.5]	2.7 [1.9–3.6]	0.95
HIV RNA			
(log_10_ copies/mL)			
blood plasma^a^	-	4.2 [3.6–4.8]	-
CSF supernatant^a^	-	4.1 [3.9–4.4]	-
HIV DNA ^e^			
(log_10_copies/million cells)			
PBMC^a^	2.2 [1.8–2.4]	3 [2.5–3.1]	0.01
CSF cell pellet^a^	3.3 [2.8–3.9]	3.3 [2.9–3.6]	0.97

^a^Data shown as median [interquartile range, IQR].

^b^For subjects on current ART.

^c^HIV RNA levels in blood plasma.

^d^One subject presented detectable levels of HIV RNA in CSF supernatant despite being suppressed in blood plasma

^e^Levels of HIV DNA in PBMC and CSF were normalized by the levels of RPP30.

EDI: estimated duration of infection; ART: antiretroviral therapy; CPE: CNS Penetration Effectiveness score. PBMC: peripheral blood mononuclear cells

### Levels of HIV DNA and RNA in CSF and blood

We analyzed a median of 147,000 PBMC (range: 138,000–157,000) and 2,590 CSF cells (range: 212–10,480) per reaction of ddPCR (based on RPP30 copy number). HIV DNA was detected in all PBMC samples with and without detectable HIV RNA viral load in plasma but at different levels. Specifically, among individuals with undetectable HIV RNA in blood plasma median HIV DNA levels were 2.2 log_10_ copies HIV per 10^6^ CD4 T cells (IQR: log_10_ of 1.8–2.4), compared to a median of 3.0 log_10_ copies of HIV DNA (IQR: 2.5–3.1) per 10^6^ CD4 cells in participants with more than 48 copies HIV RNA in blood plasma (p = 0.01, Table 1). Assuming a single copy of HIV DNA in each infected CD4 T cell, we estimate that 0.02% [IQR: 0.005–0.03%] of the entire CD4 T cell population contained HIV DNA during suppressive ART, which is in line with previous reports that shows that the frequency of HIV-infected CD4+ T cells on ART is below 1% [2].

We were able to detect HIV DNA in 18 (64%) CSF cell pellets, of which 10 (53%) samples were from subjects with undetectable HIV RNA in CSF supernatant. The median HIV DNA level in a CSF cell pellet was 3.3 log_10_ per 10^6^ cells (IQR: 2.8–3.9) among the 10 samples with undetectable HIV RNA levels in CSF, compared to 3.3 log_10_ per 10^6^ cells (IQR: 2.9–3.6) among the 8 samples with detectable HIV RNA in CSF (p = 0.15, Table 1). The calculated frequency of HIV-infected CSF cells was overall estimated as 0.2% during suppressive ART, which was approximately 10 times greater than in PBMC.

**Fig 1:**
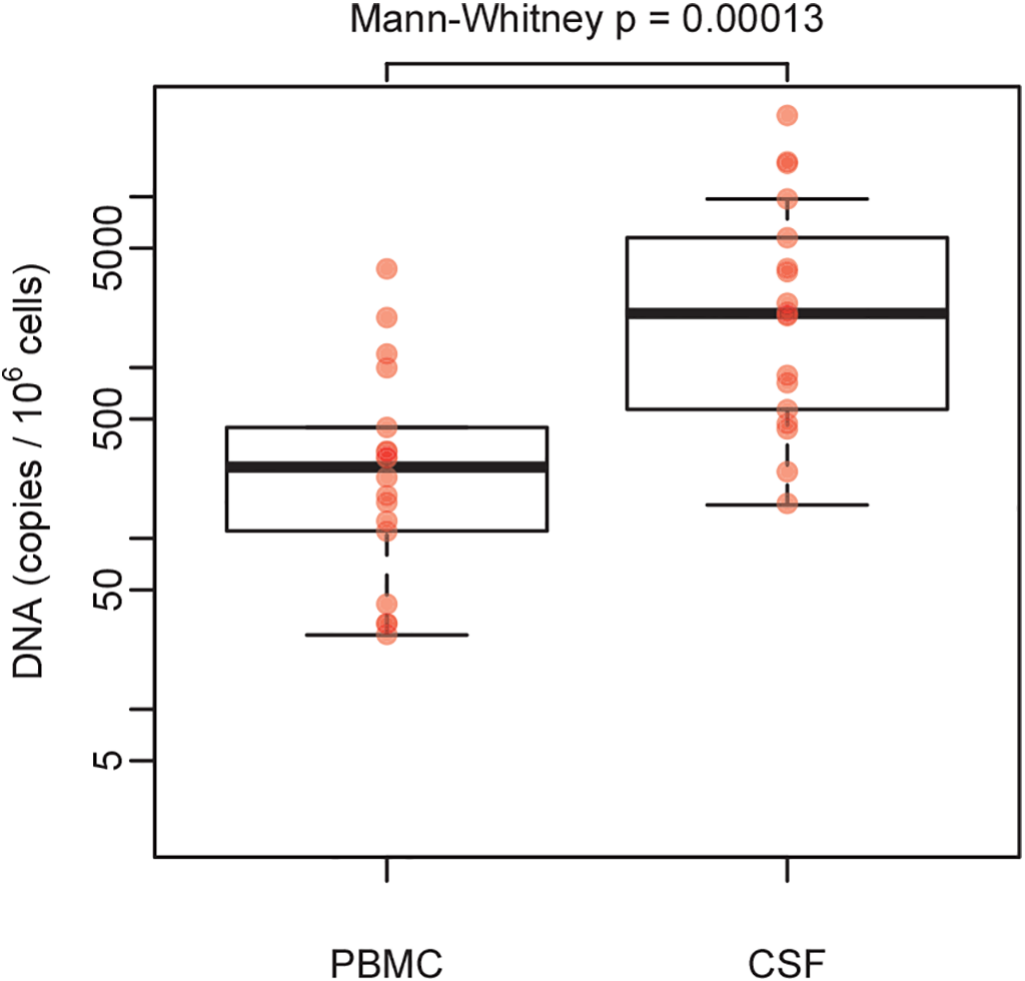
Levels of HIV DNA in PBMC and CSF cell pellets. Comparison of HIV DNA levels between PBMC and CSF cell pellets, among subjects with detectable HIV DNA in both compartments. Rank-based Mann-Whitney test p value is indicated.

When comparing HIV DNA levels between blood and CSF cellular compartments among all 28 participants, we found similar levels of HIV DNA in CSF and PBMC (2.7 log_10_ versus 2.3 log_10_, p = 0.41). However, when we repeated the analyses including only subjects with detectable HIV DNA in both compartments, levels of HIV DNA in CSF cell pellets were significantly higher compared to PBMC (3.3 log_10_ versus 2.4 log_10_, p<0.001, Fig 1). Similar results were obtained when the analysis was repeated among the subjects with suppressed HIV RNA in both blood and CSF (3.4 log_10_ versus 2.3 log_10_ p = 0.0008).

Among the 18 samples with detectable HIV DNA in CSF, the median number of calculated cells per CSF aliquot (based on (dd)PCR RPP30 values) was 3.9 cells/mm^3^ of CSF (IQR: 0.32–15.7) and the levels of HIV DNA were not significantly associated with the levels of RPP30 in CSF (τ = 0.13; p = 0.36). Similarly, levels of HIV DNA in CSF cells were not associated with CPE index (τ = 0.12; p = 0.61).

**Fig 2:**
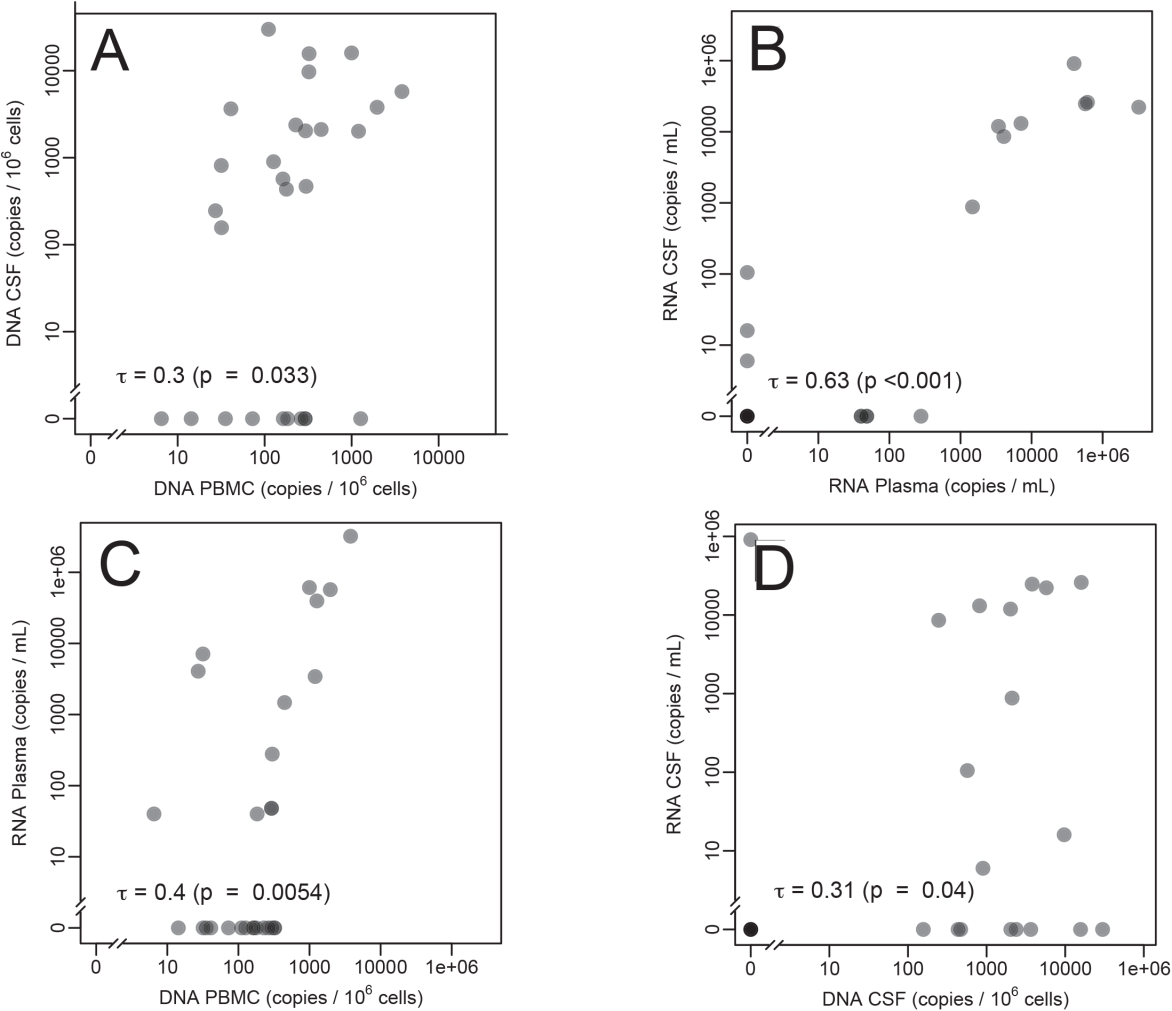
Levels of HIV DNA and HIV RNA within and between blood and CSF. (A) Association of HIV DNA levels between PBMC and CSF. (B) Association between HIV RNA levels between blood plasma and CSF. (C) Association of between HIV RNA in blood plasma and HIV DNA levels in PBMC. (D) Association of between HIV RNA and HIV DNA levels in CSF. Kendall’s rank correlation is indicated.

Next, we investigated if levels of HIV DNA in PBMC were associated with levels of HIV DNA in CSF cell pellets. Overall, there was a significant positive correlation between levels of HIV DNA measured in PBMC and CSF cell pellets (τ = 0.3; p = 0.033, Fig 2A), but 10 subjects (35.7%) had undetectable HIV DNA in their CSF cell pellets despite having high levels of HIV DNA in their PBMC. When repeating this analysis including only the 19 subjects with undetectable HIV RNA in blood plasma, this association was no more significant (τ = 0.3; p = 0.2).

We also compared the levels of HIV DNA and HIV RNA within and between blood and CSF. In agreement with previous work [13], levels of HIV RNA correlated positively between blood and CSF (τ = 0.63, p<0.001; Fig 2B) and levels of HIV DNA in PBMC were positively associated with HIV RNA levels in blood plasma (τ = 0.4, p = 0.005; Fig 2C). We also found a previously unreported, but likely expected, positive association between HIV DNA levels in CSF cells and HIV RNA in CSF supernatant (τ = 0.31, p = 0.04; Fig 2D).

**Fig 3:**
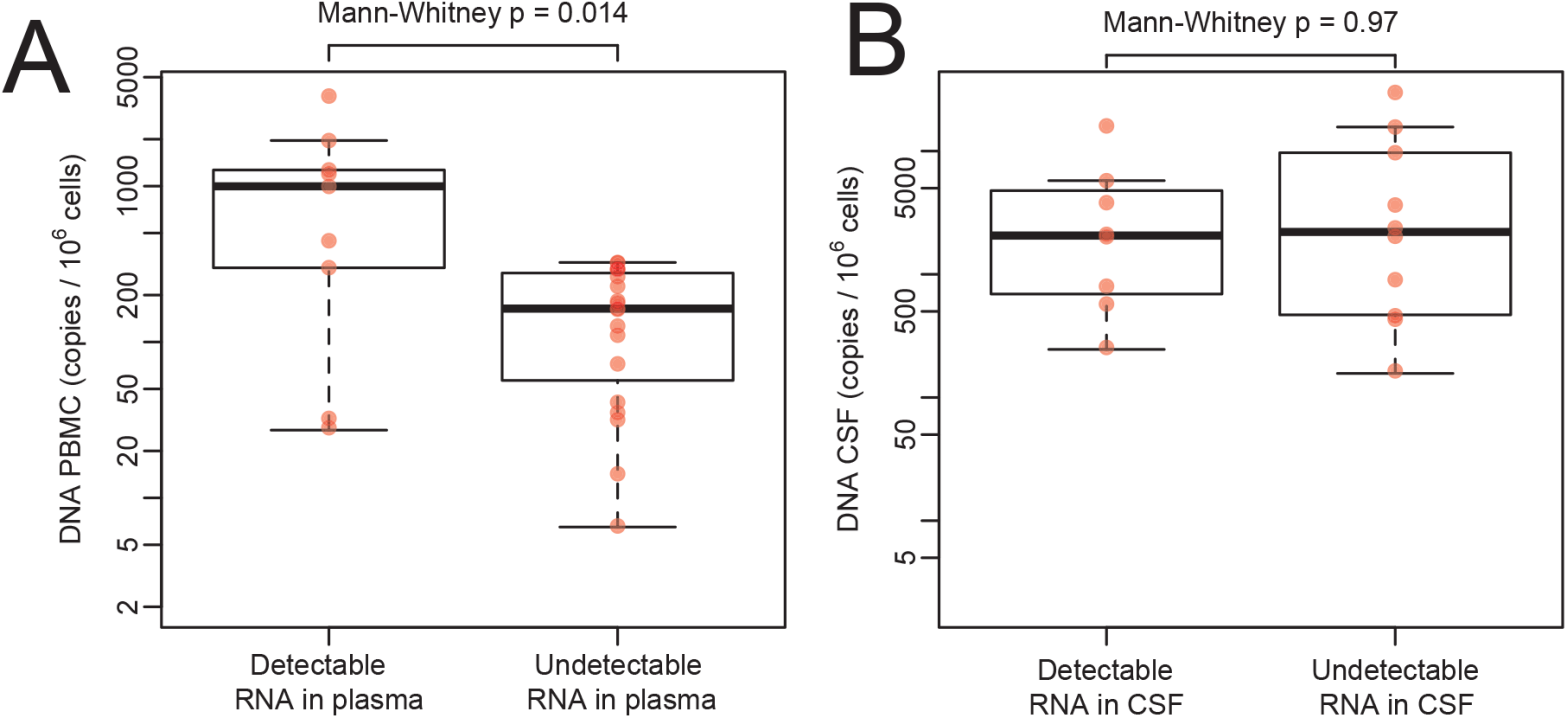
HIV DNA levels in PBMC and CSF cells between HIV RNA-suppressed and non-suppressed subjects. Comparison of HIV DNA levels in PBMC (A) and CSF pellet (B) between individuals with detectable and undetectable HIV RNA levels in blood plasma or CSF supernatant, respectively. Rank-based Mann-Whitney test p values are indicated.

To evaluate how viral suppression on ART influenced HIV DNA levels, we compared levels of HIV DNA in virally suppressed and unsuppressed participants across compartments. As might be expected, levels of HIV DNA in PBMC were significantly higher in individuals with detectable levels of HIV RNA in blood plasma when compared to subjects with undetectable HIV RNA levels (p = 0.014; Fig 3A). There was, however, no significant difference in HIV DNA levels of CSF cell pellets between participants with detectable or undetectable HIV RNA viral load in CSF supernatant, (p = 0.97 only among people with detectable HIV DNA and p = 0.14 when including the entire cohort; Fig 3B). The frequency of subjects with detectable HIV DNA in CSF was higher among those with detectable HIV RNA in CSF when compared to subjects with undetectable HIV RNA in CSF, however this was not statistically significant (89% versus 52.6%, p = 0.1).

### Rates of false-positive droplets in ddPCR for CSF cell pellets from HIV negative subjects

To determine the rates of false-positive droplets, we included 20 CSF cell pellets collected from HIV negative subjects. We observed that 3 out of 20 samples (3 replicates/sample) from HIV-negative CSF cell pellets presented one false-positive event (average of 0.33 false-positive events/replicate), while 6 out of 28 samples presented 1 single droplet/sample and 12 presented 2 or more droplets/sample (range: 2–56) in our HIV-positive cohort (S1 Fig; p = 0.0003; Mann-Whitney Test).

## Discussion

To date, very few studies have attempted to detect and quantify proviral HIV DNA in CSF, and these studies were performed using quantitative PCR (qPCR) [5,6]. Our study establishes, for the first time, the feasibility of detecting HIV DNA from frozen paucicellular CSF cell pellet samples using the (dd)PCR method [10].

While HIV DNA was detected in only a subset of CSF cellular pellets (64.2%), levels of HIV DNA were significantly higher than in blood when including only participants with detectable HIV DNA. Overall, the levels of HIV DNA in CSF were not associated with the number of input CSF leukocytes (as measured by the gene RPP30), suggesting that detectability of HIV DNA was not purely dependent from the number of cells available in each specimen. As might be expected, we found that levels of HIV DNA in PBMC were significantly lower in subjects with suppressed HIV RNA in blood plasma compared to those subjects with detectable HIV RNA. By contrast, subjects with suppressed HIV RNA levels in CSF supernatant did not have significantly lower HIV DNA levels in CSF cells than subjects with detectable HIV RNA. Previous studies have been demonstrated that the proportion of leukocytes expressing activation markers is higher in CSF than in blood [14–16], and this might reflect a greater tendency for activated leukocytes to cross the blood-CSF barrier. Since activated CD4 and monocytes are preferential targets for HIV infection [17], it is not surprising that the proportion of infected cells in CSF is higher than in blood, as observed in our study. Alternatively, although the levels of HIV DNA in CSF were not directly associated to CPE index in this dataset, we cannot exclude that this discrepancy might be a consequence of poorer ART penetration during previous regimens and slower decay of HIV DNA within CNS [18]. In support of this hypothesis, we found similar levels of HIV DNA in CSF cells between individuals with and without detectable HIV RNA in CSF. In contrast, HIV DNA was significantly lower in PBMC when HIV RNA was suppressed (compared to viremic samples), suggesting that ART is more effective in suppressing HIV DNA in PBMC compared to CSF.

We also confirmed previous reports of a positive correlation between the levels of HIV DNA and HIV RNA within blood [19]. We also observed a moderate positive correlation between the levels of HIV DNA and HIV RNA within CSF. This differs from previous a report in which there was no association among perinatally infected children presenting with HIV-1-associated encephalopathy [5]; however, this difference might be due the expected differences of viral and immunological dynamics in infants with neurological damage versus the HIV-infected adults without considerable neurological damage in our study population. We also detected a positive association between levels of HIV DNA in CSF cell pellets and PBMC. However, this association was lost when including only the 19 samples with undetectable HIV RNA in blood plasma, perhaps due to the smaller sample size or alternatively this could be a consequence of differential suppression dynamics of HIV DNA in blood compared to CSF after starting ART. This new finding supports the hypothesis that the levels of HIV DNA between blood and anatomic compartments are related during episodes of HIV RNA viremia, and is similar to another report showing a correlation between levels of HIV DNA in blood and gut in a population with mixed ART uptake in terms of HIV suppression [20].

This study has several limitations. First, the small sample size of 28 subjects (and only 19 with detectable HIV RNA) limited our ability to perform a more detailed statistical analysis and the power to detect differences and associations. Also, despite reported improved assay sensitivity [10], the low numbers of cells available for some of our frozen samples likely affected the rate of detection. To minimize loss of cells during processing and freezing, future studies will likely need to collect higher volumes of CSF and use fresh cell pellets. We also recognize that CSF represents only an approximation of the viral characteristics occurring in the CNS, but it is largely impractical to obtain *ante-mortem* brain tissues, and almost all previous studies of viral characterization between blood and CNS have used CSF as a surrogate [9,21]. Thus, the use of CSF allows at least a standard comparison for this study. Future studies evaluating HIV DNA in blood and CNS may need paired blood, CSF and brain tissues to determine the validity of using the CSF cellular pellet as a proxy for brain tissue in such studies. Because we used stored samples collected from 2000 to 2013, the impact of new classes of antiretroviral agents (e.g. integrase inhibitors) on HIV DNA levels should be evaluated separately.

Finally, since the ddPCR technology is prone to false-positive events [10,22], we included 20 CSF samples from a HIV-negative control group as no template controls. Overall, the rate of positive wells among HIV-negatives was significantly lower than in the HIV-infected cohort. In our study, we only observed single droplets in our no-template controls (in contrast to a previous study, which reported up to 3 false-positive droplets per replicate using ddPCR to quantify HIV RNA in CSF [22]. False positive events are infrequent as a percentage of all droplets analyzed, but need to be taken into careful consideration particularly when working with samples with expected low levels of viral load. These false-positive events appear randomly, are not assay or sample dependent and are not distinguishable from true positive droplets by fluorescence data. Further evaluations of these droplets to distinguish true events from false-positive events (for example sequencing of positive droplet), are not currently available as part of the standard ddPCR technology. Conservatively, the lower limit of detection could be adapted to the expected false positive threshold but this needs to be decided case by case depending on the desired level of sensitivity and specificity, the sample size and the number of tested replicates per sample. Despite these limitations, our pilot study demonstrated the feasibility of measuring HIV DNA in CSF samples with low cellular input by (dd)PCR. The evidence that cells in the CSF presents higher levels of copies of HIV DNA per million of cells than the peripheral blood needs further attention, and future studies will need to determine the proportion of HIV DNA species in CSF that is replication competent relative to that in PBMC. Similarly, our observation that suppressive ART was not associated with lower levels of HIV DNA in CSF cell samples (unlike what we observed for PBMC) deserves further investigation, to better understand the dynamics of HIV DNA reservoir in anatomic compartments during suppressive ART. A better understanding of the HIV DNA population in the CSF may help design and monitor future eradication strategies. Furthermore, because levels of HIV DNA have been associated with CNS-related complications and presence of neurocognitive impairment [4,23–25], reducing the HIV DNA reservoir in the CNS may be important in preventing or treating HIV-associated neurologic damage.

## Supporting Information

S1 Fig Frequency of positive droplets among HIV- control and HIV+ CSF cell pellet samples. Rank-based Mann-Whitney test p value is indicated.
(DOCX)
