# Supplementary material for: Comparative Analysis of Cell-Associated HIV DNA Levels in Cerebrospinal Fluid and Peripheral Blood by Droplet Digital PCR
Source: PLoS One. 2015 Oct 2;10(10):e0139510. doi: 10.1371/journal.pone.0139510 (PMC4592012; doi:10.1371/journal.pone.0139510)
Supplement: S1 Fig — Rank-based Mann-Whitney test p value is indicated. (DOCX) [file pone.0139510.s001.docx]

**Supporting S1 Fig.**
